# Hippocampal subfield and medial temporal cortical persistent activity during working memory reflects ongoing encoding

**DOI:** 10.3389/fnsys.2015.00030

**Published:** 2015-03-09

**Authors:** Rachel K. Nauer, Andrew S. Whiteman, Matthew F. Dunne, Chantal E. Stern, Karin Schon

**Affiliations:** ^1^Department of Psychological and Brain Sciences and Center for Memory and Brain, Boston University, Boston, MAUSA; ^2^Brain Plasticity and Neuroimaging Laboratory, Department of Anatomy and Neurobiology, Boston University School of Medicine, Boston, MAUSA

**Keywords:** high-resolution fMRI, working memory, medial temporal lobes, delayed matching-to-sample, hippocampus

## Abstract

Previous neuroimaging studies support a role for the medial temporal lobes in maintaining novel stimuli over brief working memory (WM) delays, and suggest delay period activity predicts subsequent memory. Additionally, slice recording studies have demonstrated neuronal persistent spiking in entorhinal cortex, perirhinal cortex (PrC), and hippocampus (CA1, CA3, subiculum). These data have led to computational models that suggest persistent spiking in parahippocampal regions could sustain neuronal representations of sensory information over many seconds. This mechanism may support both WM maintenance and encoding of information into long term episodic memory. The goal of the current study was to use high-resolution fMRI to elucidate the contributions of the MTL cortices and hippocampal subfields to WM maintenance as it relates to later episodic recognition memory. We scanned participants while they performed a delayed match to sample task with novel scene stimuli, and assessed their memory for these scenes post-scan. We hypothesized stimulus-driven activation that persists into the delay period—a putative correlate of persistent spiking—would predict later recognition memory. Our results suggest sample and delay period activation in the parahippocampal cortex (PHC), PrC, and subiculum (extending into DG/CA3 and CA1) was linearly related to increases in subsequent memory strength. These data extend previous neuroimaging studies that have constrained their analysis to either the sample or delay period by modeling these together as one continuous ongoing encoding process, and support computational frameworks that predict persistent activity underlies both WM and episodic encoding.

## Introduction

Converging evidence from human neuroimaging (Fernández et al., 1999; Kirchhoff et al., 2000; Preston et al., 2010) and animal studies (Zola-Morgan and Squire, 1986; Beason-Held et al., 1999; for review see: Eichenbaum, 2000; Squire et al., 2004) have implicated the hippocampus (HC), parahippocampal cortex (PHC), entorhinal cortex (EC), and perirhinal cortex (PrC) as critical for long-term episodic encoding. In some of these regions, most notably PrC (but also PHC and hippocampus), subjective memory strength appears to modulate encoding-related activity (Gonsalves et al., 2005; Montaldi et al., 2006; Kirwan et al., 2008; Shrager et al., 2008; Song et al., 2011). Furthermore, fMRI studies indicate these medial temporal lobe (MTL) structures show greater activation for novel compared to familiar stimuli during working memory (WM) tasks (Ranganath and D’Esposito, 2001; Stern et al., 2001). Specifically, delay period activity during WM tasks predicts both immediate (Olsen et al., 2009) and longer-term subsequent memory (Schon et al., 2004, 2005; Ranganath et al., 2005; Nichols et al., 2006). However, the distinct contributions of hippocampal subfields during WM tasks and how WM task related activity in these regions may contribute to long-term subsequent memory have not been examined with human neuroimaging.

Computational models have proposed persistent spiking as a mechanism that could support successful encoding of long-term episodic memories (Hasselmo et al., 2002; McGaughy et al., 2005; Hasselmo and Brandon, 2008). Electrophysiological and single-unit slice recording studies have provided evidence for neurons that, once sufficiently depolarized, can sustain persistent firing activity for up to several minutes after cessation of the input stimulus (e.g., Jochems and Yoshida, 2013). In particular, within the hippocampal memory system, recent work has shown that subsets of neurons in the EC, PrC, and hippocampal subfields CA1 and CA3 are known to possess these characteristics (Klink and Alonso, 1997; Young et al., 1997; Tahvildari et al., 2007; Yoshida et al., 2008; Navaroli et al., 2012; Jochems and Yoshida, 2013; Knauer et al., 2013). These data have led to the idea persistent spiking may act as an episodic memory buffer, supporting long-term encoding of information past the duration of a sensory event, and suggest a neural mechanism sufficient for short-term maintenance of novel stimuli (Lisman and Idiart, 1995; Jensen and Lisman, 1996; Fransén et al., 2002; Koene et al., 2003; McGaughy et al., 2005). In support of this hypothesis, electrophysiological recordings in awake, behaving monkeys and rats have shown persistent, stimulus selective activity in EC neurons during the delay period of delayed (non-) match to sample tasks (Suzuki et al., 1997; Young et al., 1997).

We hypothesized persistent spiking (and/or other mechanisms of short-term maintenance, see Ongoing Encoding is Consistent with Persistent Spiking Mechanisms in MTL) may underlie a process of encoding which extends from initial stimulus presentation through an indeterminate period of time when a stimulus is absent, a process we refer to as ongoing encoding. Anatomically, the EC relays incoming information to the hippocampus, and has direct projections to the dentate gyrus (DG), CA1, and CA3 subfields of the hippocampus (Van Hoesen and Pandya, 1975; Witter et al., 1989). PrC and PHC preferentially project to lateral and medial EC, respectively, as well as have direct projections to the hippocampus (see Witter et al., 2000 for a review). Animal single-unit recordings from hippocampal subregions CA1 (Knauer et al., 2013) and CA3 (Jochems and Yoshida, 2013), as well as in PrC (Young et al., 1997; Navaroli et al., 2012) and EC in both rodents (Klink and Alonso, 1997; Tahvildari et al., 2007; Yoshida et al., 2008) and primates (Suzuki et al., 1997) lead us to postulate that increased activity persisting into the delay period in the absence of continued stimulus input may be reflective of persistent spiking mechanisms.

Guided by both known anatomical connections and computational theories, we used high-resolution fMRI to elucidate the contributions of the hippocampal subfields and MTL cortices during maintenance of visual scene stimuli in a delayed match-to-sample (DMS) task. Additionally we sought to determine whether persistent fMRI activity in MTL regions predicts subsequent long-term recognition memory strength. If modulation of the blood–oxygen level dependent (BOLD) response across the sample period extending into delay is related to an increase in subsequent memory strength, this would provide evidence for ongoing encoding in these regions. We predicted the magnitude of sustained activation in the DG/CA3 and CA1 subregions of the hippocampus, as well as throughout the parahippocampal cortical regions would reflect subsequent long-term memory. Results suggest activity in these regions that persists into the delay period is linked to subsequent memory strength.

## Materials and Methods

### Participants

Thirty-four healthy individuals were recruited from the Boston University student community. Participants were native English speakers or bilingual with no reported neurological or psychiatric history, and had normal or corrected-to-normal vision. All participants gave signed, informed consent before participating in this study, and all protocols were approved by the Boston University Charles River Campus Institutional Review Board and adhered to the Code of Ethics of the World Medical Association. Six participants were excluded from analyses: three due to excess motion during fMRI scanning and three due to equipment malfunction, resulting in the inclusion of *N* = 28 participants (mean age 20.9 ± 2.2 years; 11 males) for data analysis.

### Behavioral Procedure

During functional scanning, participants performed an adapted DMS paradigm using trial-unique complex visual outdoor scenes to assess maintenance of visual stimuli during short delays (Schon et al., 2004, 2005). Approximately 15 min following the scanning session, participants performed a surprise subsequent memory test (SMT) to assess successful encoding of the DMS task stimuli. The stimulus set for both the DMS and SMT tasks were selected from 288 trial unique, but content similar complex visual scenes (Stern et al., 1996, 2001; Schon et al., 2004, 2005; Whiteman et al., 2014). Participants viewed 144 scenes during the DMS task, and the remaining 144 stimuli served as unfamiliar lures and were seen during the SMT only.

**FIGURE 1:**
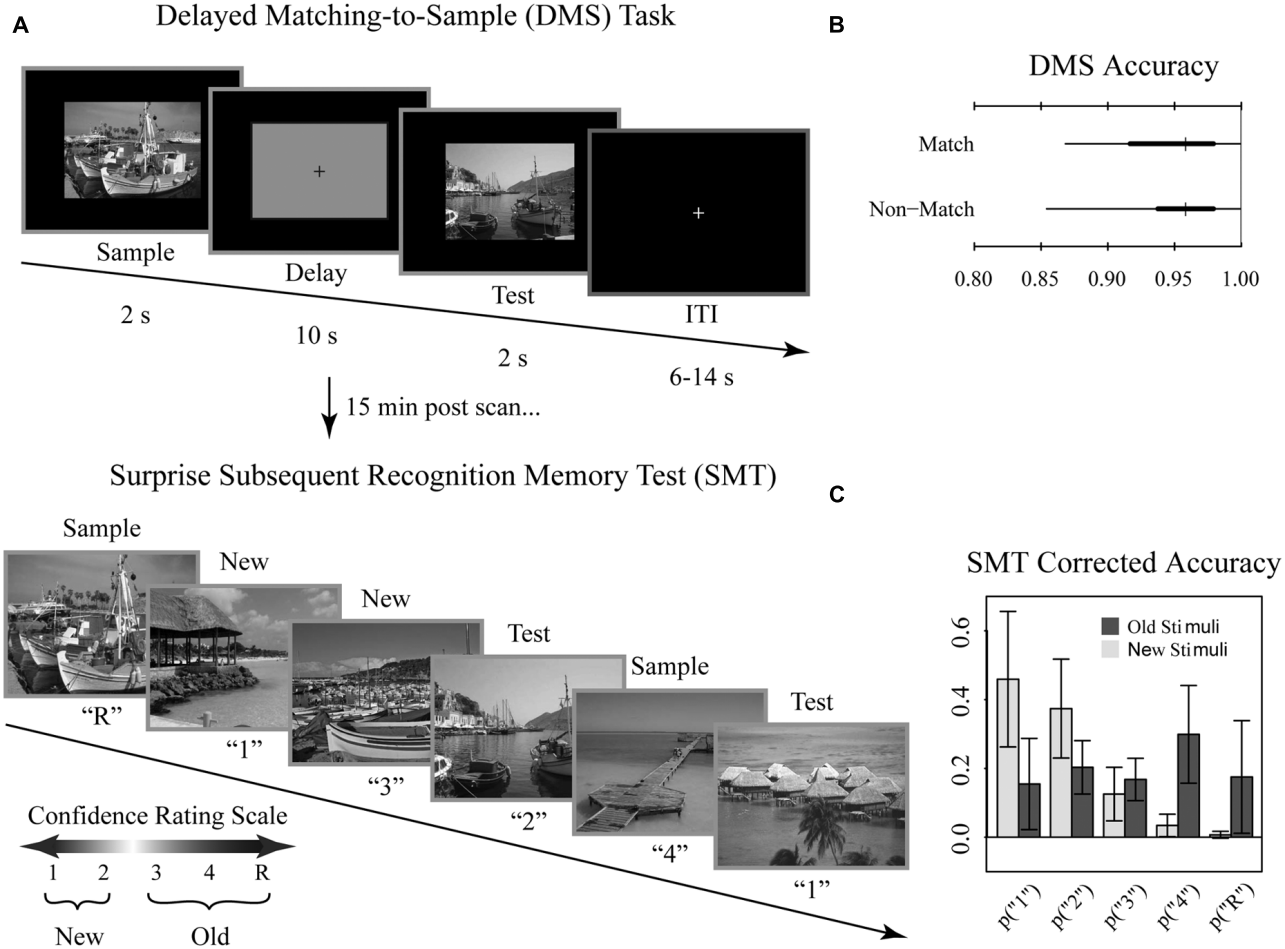
**Tasks and behavioral results.** Recognition memory task adapted from Schon et al. (2004). **(A)** Participants were first shown a series of 144 randomized, trial unique but content similar outdoor scenes in the context of a delayed match to sample (DMS) working memory (WM) task during fMRI scanning. Approximately 15 min after completion of the fMRI scanning session, participants were administered a surprise subsequent memory test (SMT) where they were shown all 144 DMS images, plus 144 lure images, and asked to rate their recognition confidence. Participants were blind to the ratio of old to new images on the SMT. **(B)** Overall DMS task accuracy, separated by match and non-match trials. Ticks, thick lines, and thin lines show medians, 50% intervals, and 95% intervals, respectively. **(C)** SMT response distributions for old and lure stimuli, separated by confidence rating. Error bars show SD.

Participants performed the DMS task across eight runs comprised of 12 trials per run, for a total of 96 trials. Each DMS trial consisted of a 2 s initial scene presentation (sample), followed by a 10 s delay period, followed by a 2 s presentation of a visual scene (test; **Figure 1A**). During the delay, subjects viewed a grayscale box maintaining the same dimensions as the visual scenes with a black fixation cross at the center. Trials were separated by a variable length (6, 10, or 14 s) inter-trial-interval to introduce temporal jitter. During the test phase, participants were asked to make a yes-no judgment of whether the test scene was a match to the sample scene for that trial. Trials were evenly split into match and non-match conditions. Match and non-match trials were pseudo-randomized for each run and participant. Immediately prior to entry to the scanner, participants performed short practice runs to familiarize them with the task.

Approximately 15 min after the scanning session, participants performed a self-paced surprise SMT to examine incidental encoding of the DMS stimuli. For each image, participants were asked to rate their recognition memory strength using a confidence rating scale to distinguish all 144 DMS scenes from an equal number of content similar lures. Participants were blind to the ratio of old to new images. The scale was as follows: (1) high confidence new, (2) low confidence new, (3) low confidence old, (4) high confidence old, (R) high confidence old with additional contextual memory for the stimuli. Participants were instructed to select the R response only when additional contextual information was recollected (e.g., subject remembered during which run they had encountered the stimulus or whether the scene was a match or non-match during the DMS task; subject recalled a particular thought they had during that stimulus presentation, etc.). Note that, while we discuss “memory strength” throughout as it relates to this scale, we take a neutral stance on the debate between dual process vs unequal variance signal detection. Here, we define memory strength as how resolutely encoded information was subsequently endorsed as remembered. Stimuli were presented and responses were recorded using E-Prime 2 (Psychology Software Tools, Inc., Pittsburgh, PA).

### Behavioral Performance Analysis

Behavioral performance was analyzed using R 2.15.3 for both DMS and SMT task performance. Performance on the DMS task was assessed as percent correct. For the SMT analysis, we included responses from correct DMS trials only. SMT performance data were scored as corrected accuracy (proportion of true positives – proportion of false positives).

### MRI Data Acquisition

**FIGURE 2:**
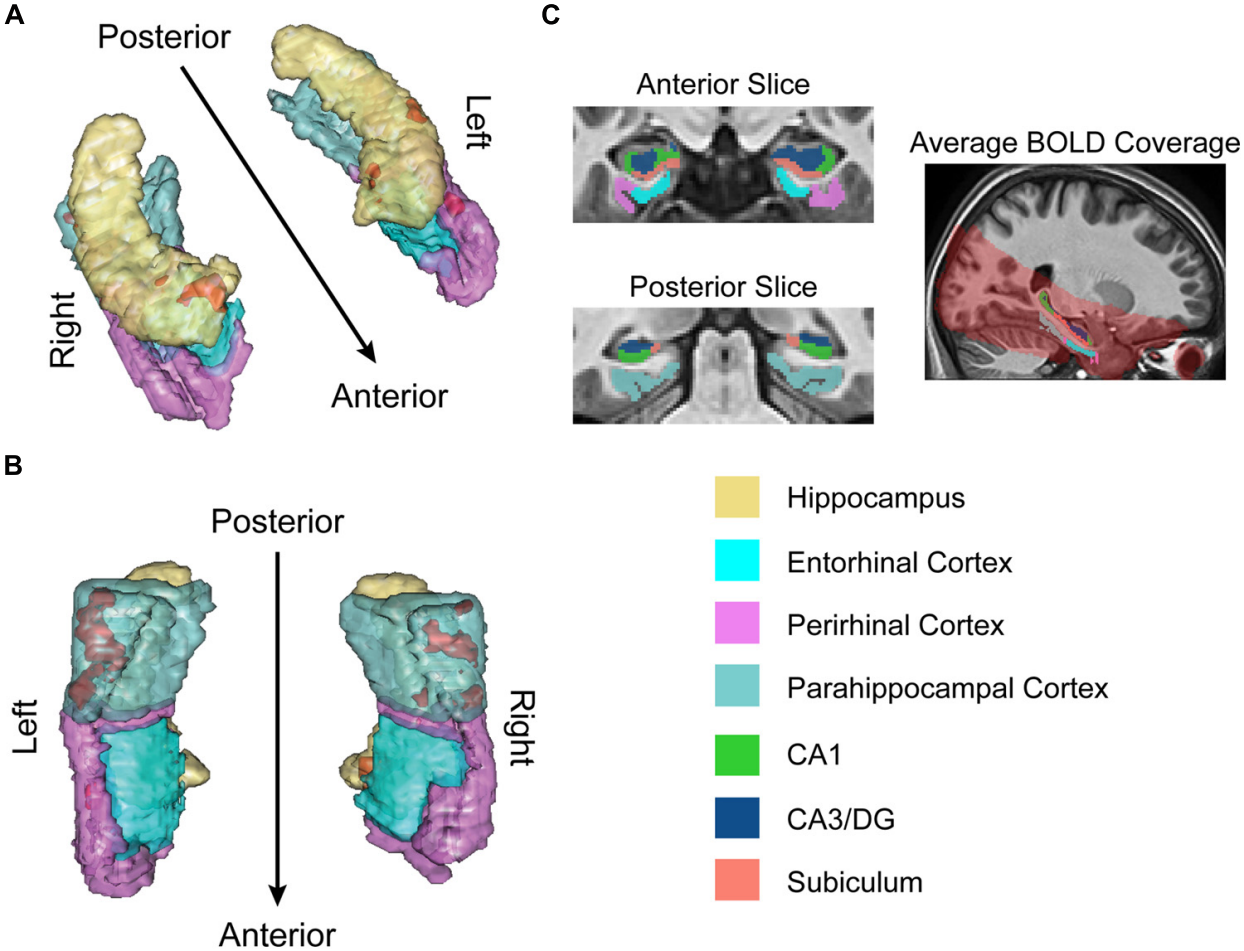
**fMRI results for ongoing encoding (*sample + delay* model without signal decay).** Preliminary analysis of a putative encoding epoch (sample + delay periods from DMS task; see **Figure 1**) shows regions where increased activity during DMS task performance was linearly related to stronger episodic memory at later test. Results are shown in red within 3D renderings of our regions of interest (ROIs) from both dorsal **(A)** and ventral **(B)** perspectives. Part **(C)** shows example ROI tracings from anterior and posterior slices, and the region of BOLD signal coverage averaged across participants. Note the apparent lack of major signal dropout in EC. Figures were made using functionality from the R *rgl* and *misc3d* packages (Feng and Tierney, 2008; Adler et al., 2014).

Structural and functional imaging data were collected on a 3-Tesla Philips Achieva scanner with an 8-channel SENSE head coil at the Boston University Center for Biomedical Imaging in Boston, MA, USA. A high-resolution structural T1-weighted magnetization prepared rapid acquisition gradient echo (MP-RAGE) structural scan was acquired for each participant (SENSitivity Encoding P reduction: 1.5, S reduction: 2; TR = 6.8 ms, TE = 3.1 ms, flip angle = 9°, Field of View = 25 cm, Matrix Size = 256 × 254, 150 slices, resolution = 0.98 mm × 0.98 mm × 1.22 mm). For each participant, a total of 144 functional BOLD volumes were acquired for each of the 8 scanning runs, resulting in 1152 functional images. BOLD images were acquired using a high-resolution T2^∗^-sensitive echo planar imaging (EPI) sequence with an in plane acquisition resolution of 1.5 mm^2^ and slice thickness of 1.5 mm (TR = 2 s; TE = 26 ms; flip angle = 70°; Field of View = 180 × 240; matrix size = 120 × 160; 20–21 slices per volume; SENSE P reduction = 2.5). Additionally, we obtained a T1-weighted inversion recovery image for each subject (TR = 3374 ms; TE = 15 ms; flip angle = 120°; Field of View = 183 × 230; matrix size = 256 × 157; resolution = 0.9 mm × 1.1 mm × 5 mm; 24 slices). BOLD and T1-weighted IR scans were aligned parallel to the long axis of the hippocampus to ensure inclusion of the hippocampal subfields (subiculum, CA1, CA3/DG) and the MTL cortices (perirhinal, entorhinal, parahippocampal) and acquired in the axial plane. Region of BOLD coverage averaged across all participants shown in **Figure 2C**.

### fMRI Image Processing

We performed cross-participant alignment of neuroimaging data in two steps: (i) a standard intensity-based whole brain alignment described in this section and, (ii) a more precise, label-based alignment using manually segmented regions of interest (ROIs) described in Section “fMRI Data Analysis,” below. The first step in this process was implemented using the SPM8 software package (Statistical Parametric Mapping, Wellcome Department of Cognitive Neurology, London, UK), running on a MATLAB 8.1 (R2013a) platform (The MathWorks, Inc., Natick, MA, USA). All BOLD images were reoriented to adopt the point of origin [i.e., coordinate xyz = (0, 0, 0)] at a position 8 mm ventral to the anterior commissure. Motion parameters were estimated in SPM8, and slice artifacts were detected and repaired with the ArtRepair toolbox^1^. BOLD images were motion corrected by aligning them to the first image within a series. Next, BOLD and MP-RAGE scans were co-registered with the T1-weighted IR image. This co-registration step maximized the mutual information between the partial BOLD images and the high-resolution, whole-brain T1-MPRAGE image. In addition, MP-RAGE scans were segmented into gray matter, white matter, and CSF probability maps. This step produced a bias-corrected anatomical image. In addition, this step generated spatial normalization parameters using SPM’s default tissue probability maps. Anatomical and functional images were then normalized to MNI (Montreal Neurological Institute) space using the spatial normalization parameters generated during the segmentation step and resampled to a resolution of 1 mm × 1 mm × 1 mm isotropic voxels, and BOLD images were spatially smoothed using a 3 mm^3^ Gaussian filter. Precise cross-participant alignment of specific ROIs was achieved employing the ROI-ANTs technique described in the following section.

### ROI Based Cross-Participant Alignment Using Advanced Normalization Tools

For optimal between subjects co-registration of the anatomical substructures of the hippocampus and MTL cortices, we employed a region of interest-based method (Stark and Okado, 2003; Yassa and Stark, 2009). ROIs included the PHC, PrC, and EC, and the hippocampal subfields CA3/DG, CA1, and subiculum (with the current resolution of *in vivo* human neuroimaging techniques, it is not yet possible to delineate CA3 from the DG reliably). All ROIs were defined anatomically and sectioned manually in ITK-SNAP^2^ (Yushkevich et al., 2006). To maintain consistency, one researcher (MD) completed all ROI tracings which were subsequently verified by a second researcher (AW). Boundaries for the MTL cortices and hippocampus were defined using previously published guidelines (Pruessner et al., 2000, 2002). All boundaries for hippocampal subfields were defined using the Duvernoy (2005) atlas and methods described in previous studies (Kirwan et al., 2007; Newmark et al., 2013). Briefly, borders for the PrC and EC were defined anteriorly with the appearance of the collateral sulcus (CS) and posteriorly with the disappearance of the intralimbic gyrus. The lateral border of PrC extended to the crest of the lateral bank of CS. When the EC was present, the midpoint of the medial bank of the CS formed the medial border of PrC and lateral border of the EC. The medial border of EC (and PrC when EC not present) was amygdala if present or hippocampus if not. The anterior border of the PHC began 2 mm posterior to the caudal end of EC; PHC continued posteriorly until the disappearance of hippocampal tail. The lateral border of PHC was the crest of the lateral bank of the CS and the medial border was hippocampus until appearance of calcarine sulcus—here the inferior edge of calcarine sulcus became the medial border.

Deformation fields to warp each subject’s traced hippocampi and cortices to a template space were then estimated using the ANTs^3^ (Advanced Normalization Tools) software package, a state-of-the-art medical image registration and segmentation program, and specifically the SyN algorithm (Avants et al., 2008). All ANTs components are capable of leveraging multivariate image features as well as expert knowledge in order to learn the best segmentation strategy available for each individual image (Avants et al., 2011; Wang et al., 2011). This flexibility has led to ANTs winning several internationally recognized medical image processing challenges (Klein et al., 2009; Wang and Yushkevich, 2013; Menze et al., 2014), providing relative validation for the method. Once the algorithm generated the deformation field images for each participant we applied them to all BOLD images to warp them into the common template space. The template brain was also created from a subset of 12 representative T1-MPRAGE images using ANTs routines.

### fMRI Data Analysis

Once all BOLD scans were aligned across participants with ROI-ANTs, we converted the data into an R readable format using custom software written for MATLAB and SPM8. All further analysis was performed using R 2.15.3 (R Development Core Team, 2012).

For the within-subject first level analysis, we fit a voxel-wise standard least squares regression model to the time series data (*k* = 14,406 voxels in the MTL). Our goal was to estimate the average increase in BOLD signal when stimuli are later remembered or forgotten. Because we hypothesized that persistent spiking mechanisms (or short-term maintenance) in hippocampus and MTL cortex could result in elevated activity sustained past stimulus cessation, we began by modeling sample and delay periods together as part of a single encoding epoch, here termed “ongoing encoding” (see Introduction), and focused primarily on this task component. We modeled the ongoing encoding period across match and non-match trials including both correct and incorrect DMS trials. We therefore created a set of up to 10 regressors of interest per subject: a *sample + delay* regressor and a *test* regressor for each of the five [1–4, R] responses during the post-scan SMT (*N* = 7 subjects did not use the full set of responses and do not have the full set of 10 regressors of interest). We then convolved all regressors of interest with a canonical hemodynamic response function (double gamma). The coefficient on the completed *sample + delay + R* regressor, for example, can therefore be interpreted as the increase in BOLD signal over the sample and delay period (i.e., assumed “ongoing encoding” period) averaged over all instances of stimuli subsequently labeled “*R.”* We did not explicitly model the inter-trial-interval period; this task component is absorbed by the intercept terms in the model (see below).

Our within-subject models also included nuisance regressors for features of the BOLD signal not related to cognitive task components. In particular, we divided our intercept into a set of eight factors representing differences in run-to-run signal baseline. We included six motion regressors using the x-, y-, and z-translations, and pitch-, roll-, and yaw-rotations determined during the motion correction step (see ROI Based Cross-Participant Alignment Using Advanced Normalization Tools, above); we also modeled interactions between each of these and our run factor. Finally, we modeled scanner drift as a cubic polynomial: we setup a regressor for linear drift (coded as a sequence from -72.5 to 72.5 in increments of 1, repeated for each of the eight runs; 144 time points per run) and also included this drift regressor squared and cubed separately in the model for added flexibility in fitting the quasi-linear trend. We also modeled the interactions between our drift regressors and our run factor since the effect of scanner drift differs slightly between runs. All other models we fit are minor variations on this preliminary analysis and are described in the results section (see Imaging Results).

At the group level, we were interested in exploring whether increases in encoding related activity reliably predicted subsequent memory strength across participants. To this end, we set up a model for the set of *sample + delay* coefficients from the first level analysis, using weighted least squares to account for the uncertainty in these estimates (data points weighted by the inverse of the coefficient variance estimates from the first-level models). For this analysis, the group level design matrix included a single predictor for memory strength on a linear scale (coded -2 for a “1” response, 2 for an “R” response, and increments of 1 in between). The estimated coefficient on this regressor can be interpreted as the average increase, across all participants, in change in BOLD signal per point increase on the memory strength scale (for example, moving from a response of “3” to a response of “4”) or, alternatively, how BOLD signal change is parametrically modulated by memory strength. Because we have up to five data points per participant, we split our intercept up into a set of 28 factors to model subject-to-subject variation in mean BOLD signal change (*N* = 28 participants).

Many reports of high-resolution fMRI studies do not correct for multiplicity of null-hypothesis tests, possibly because hypotheses are frequently defined anatomically (here, we excluded data from all regions outside the hippocampus and parahippocampal regions). For practical purposes, we employ a more moderate two-tailed alpha level of 0.01, uncorrected. We report and visualize all our results based on this threshold. To provide a rigorous comparison point, we also report inference corrected to a family wise error rate of α < 0.05 based on a permutation test for the peak voxel in an image (Nichols and Holmes, 2002). Permutation tests are more powerful than other family wise error correction methods (e.g., FDR, Bonferroni, etc.), and are always valid as way to correct inference on any test statistic because they make very few assumptions about the data (Nichols and Holmes, 2002). We generated a null distribution for our second level *t*-statistic images by refitting the model to 1000 random permutations of the order of our response data (order kept constant for all voxels within one permutation). We then found the maximum *t*-statistic for each permutation image, thereby generating a distribution for the maximal *t*-statistic we would expect to find by chance. A family wise error corrected *P*-value for an observed *t*-statistic derived through this permutation test can then be defined as the proportion of null distribution maximal *t*-statistics greater than the observed value.

## Results

### Behavioral Results

Overall, participants performed near ceiling on the DMS task (median accuracy 95%; range 85–100%; chance 50%). DMS performance did not differ for match and non-match trials (**Figure 1B**). For the subsequent memory task, we determined average corrected accuracy by subtracting the false positive rate from true positive rate, yielding a median accuracy score of 48% (range 26–68%; **Figure 1C**). Given that for this measure of corrected accuracy chance performance is 0%, subjects exhibited reliable differentiation between old and new stimuli.

### Imaging Results

We began our analysis with the goal to test the hypothesis that activity driven by sample stimulus processing would persist across the delay period with no signal decay. Furthermore, we predicted the magnitude of this sustained activation would be related to subsequent episodic memory strength, lending indirect support to our hypothesis that continued activity reflects ongoing encoding.

The results of this analysis (summarized in **Figure 2**) suggested activity in the MTL was, on average, elevated across sample and delay. Furthermore, the degree of this activation was linearly related to subsequent memory strength. Subsequent memory related activations were dominated by large, bilateral clusters in PHC (**Figure 2B**; *k_left_* = 306; *k_right_* = 113), consistent with our previous work (Schon et al., 2004, 2005). We also observed somewhat smaller, yet substantial clusters in the right PrC (**Figure 2A**; *k* = 55) and right subiculum extending into DG/CA3 and CA1 (**Figure 2A**; *k* = 99).

**FIGURE 3:**
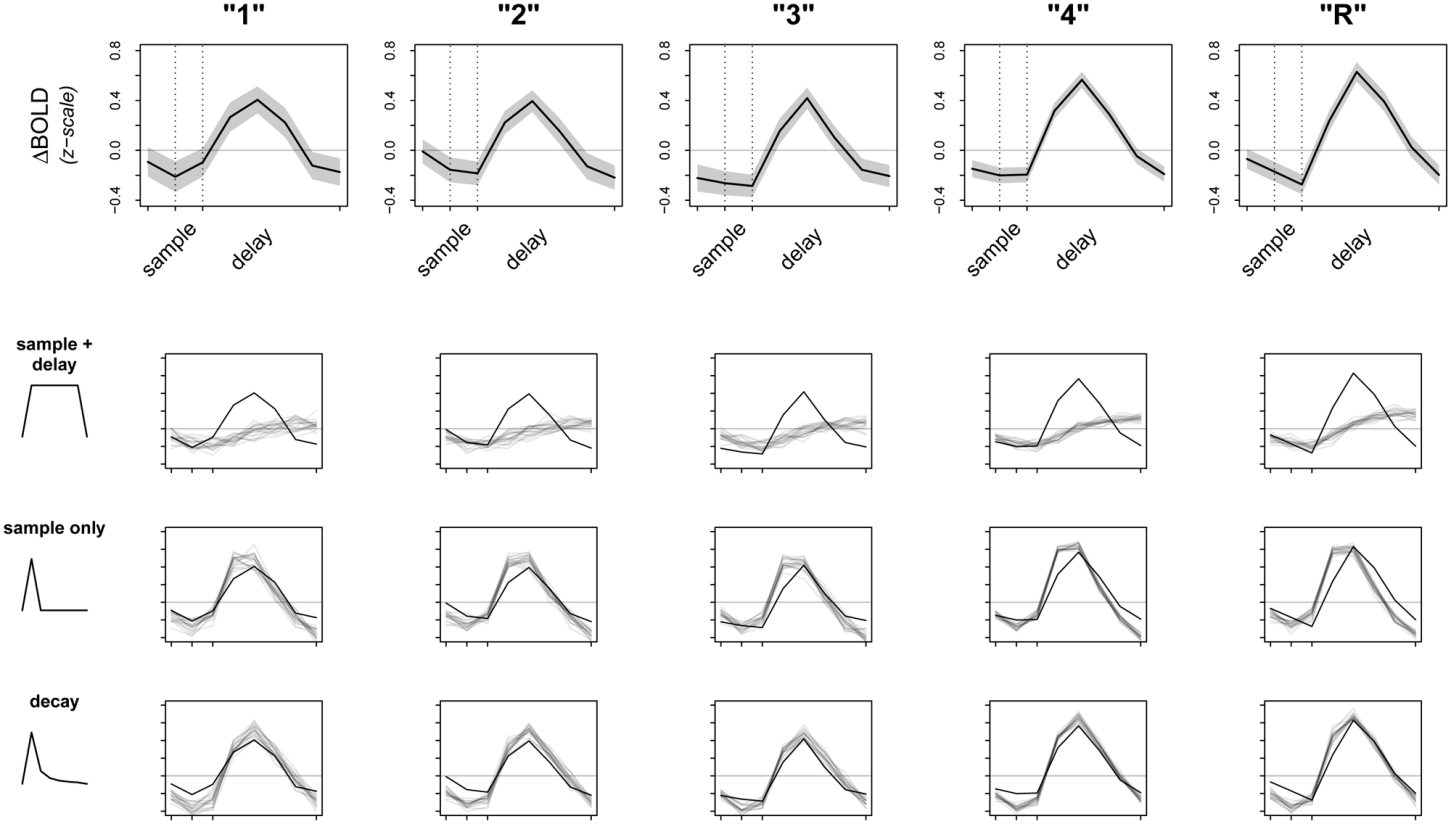
**Observed data and model fit of three encoding models.** The top row shows the raw data from a 5 mm sphere around the peak voxel in L. PHC, averaged over all participants and all trials. Whole time series are *z*-scored within participant prior to averaging to transform the data to a common scale; the horizontal gray line represents the mean of the raw time series. Dotted lines correspond to the onsets of sample and delay periods, respectively. Note that due to the sluggishness of the hemodynamic response, signal from a given time point is shifted later by ∼6 s. The bottom three rows test the fits (gray lines) of three separate models against the observed data (black lines). Dark gray lines are averages from 20 random posterior predictive simulations, depicting what we would expect the data to look like if the underlying model were true. Regions where the observed data do not overlap with the simulations represent discrepancy between observed data and model fit.

Given these preliminary results, we then sought to check the fit of our model. A primary concern was that the raw signal could be driven entirely by activity during the sample period, which, although informative, would be contrary to our ongoing encoding hypothesis. If this were true, averaging over sample and delay together could still appear as an increase in overall activation when we fit our model. To test this alternate hypothesis, we revised our original model to average over activity due to sample period only (instead of *sample + delay*) and refit the model to the data. We then attempted to falsify both *sample + delay* and *sample only* models. To do this, we used posterior predictive simulation (Box, 1980; Rubin, 1984; Gelman and Hill, 2007) to compare the observed data to simulations of what we would expect the data to look like if our models were true. Results of this analysis are presented in **Figure 3**, with the raw data (means ± 2⋅SE) from left PHC shown in the top row, and results from the *sample + delay* and *sample only* models shown in the second and third rows. The smaller graphs in **Figure 3** show the results of a vector test statistic, with regions of systematic discrepancies between observed and simulated data representing features of the data that are not captured well by the model. The ROI data shown in **Figure 3** were averaged over a 5 mm sphere surrounding the peak parahippocampal voxel from our preliminary *sample + delay* analysis. Within our other ROIs (right PrC, CA3/DG, CA1, subiculum) we found the data surrounding other regional maxima looked strikingly similar (data not shown).

Perhaps not surprisingly, both models (*sample + delay* and *sample* only) appear to fit the data poorly, especially as subsequent memory strength increases (along the columns of **Figure 3**). Therefore, we revised our original hypotheses and fit a third model that incorporated an initial response to the sample stimulus, followed by a slow decay in activity throughout the whole delay period. While we do not have a specific hypothesis for the shape of the decay function, the model was loosely based on electrophysiological data showing firing rates in persistent spiking neurons decline when direct stimulation is turned off (e.g., Jochems and Yoshida, 2013). For the model we used, the decay is proportional to the inverse square of the number of time points across sample and delay periods [e.g., the pre-convolution values for the first three time points—sample TR, first two TRs of delay—are: (1^-2^, 2^-2^, 3^-2^, …), and so on]. The fit of this model is summarized in the last row of **Figure 3**. Although there are still regions of discrepancy, this model represents a major improvement over our two earlier models. In particular, the decay model predicted the rise and fall of the BOLD response across sample and delay periods, as well as the three-point pinnacle in the middle of the signal, much better than do the other two models (**Figure 3**). These features of the data could not plausibly have arisen under either the *sample + delay* or *sample only* models. From this result we conclude evidence of activity persisting into the delay period in the absence of continued sensory input.

**FIGURE 4:**
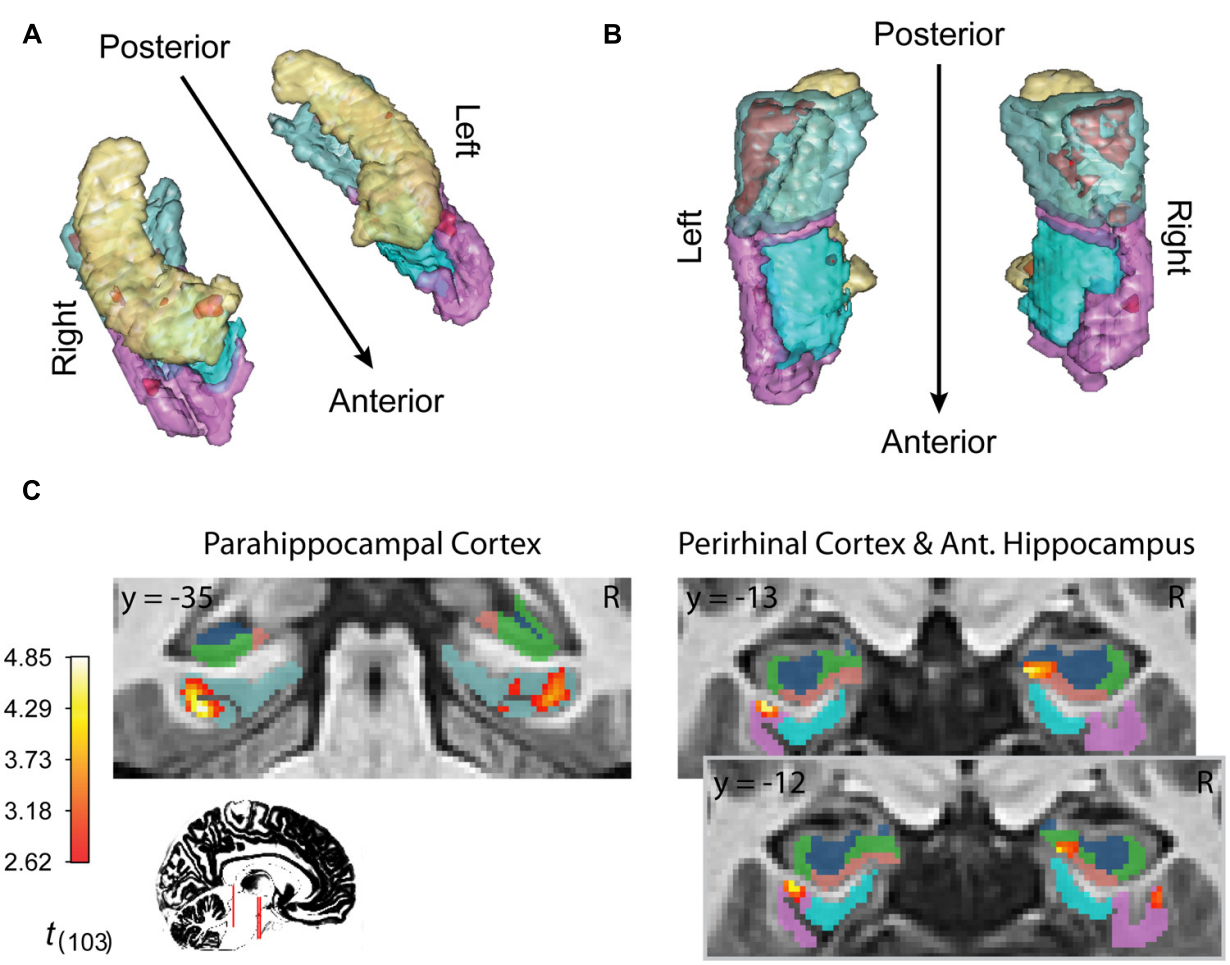
**fMRI results for ongoing encoding with slow decay.** Main results from slow decay model analysis showing regions where BOLD response is initiated at the onset of the sample stimulus (see **Figure 1**) and persists into the delay period, slowly decaying with time (see **Figure 3**). The magnitude of this activity was linearly related to subsequent strength of episodic memory. Results are shown in red within 3D renderings of our ROIs from both dorsal **(A)** and ventral **(B)** perspectives. Individual slices **(C)** follow **Table 1** in showing the locations of peak voxels within main clusters. Figures were made using functionality from the R *rgl* and *misc3d* packages (Feng and Tierney, 2008; Adler et al., 2014).

Finally, inference from this decay model as it relates to subsequent memory strength at the group level is presented in **Figure 4**; **Table 1**. We considered the results of this model above the others when drawing conclusions from the present study. Very large regions of PHC appeared to follow this model where magnitude of the sample and decaying delay period signal linearly predicted greater subsequent memory strength (clusters comprise ∼22 and 17%, respectively, of the total volume of the left and right PHC; **Figure 4B**). A relatively large region of the right anterior hippocampus also seemed to follow the model (**Figure 4A**). The peak of this cluster was in the subiculum, but the activation cluster also extended into DG/CA3 and to a lesser extent into CA1 (**Figure 4C**; **Table 1**). We do not characterize the location of this result further as reliable delineation of subfields in anterior hippocampus is particularly difficult. We also found two smaller clusters in left and right PrC (**Figure 4C**; **Table 1**). At a two-tailed α < 0.01 threshold, a total of 957 voxels were labeled as potentially significant findings. Of these, *t*-statistics in 934 voxels (98%) were positive in sign and only 23 voxels were negative (2%—likely reflecting false positives). Finally, at our strict family wise error corrected threshold, two adjacent voxels in left PHC were labeled as potentially significant findings. Based on the permutation distribution, the probability of finding two or more voxels labeled at this threshold by chance is approximately 2%. These results provide strong evidence that MTL signal during WM performance is associated with later scene recognition strength.

**Table 1:** Anatomical summary of results from decay model of ongoing encoding.

Region	Peak coordinates	Peak *t*_(103)_	Cluster size *k*
L. parahippocampal cortex (PHC)	(31, -35, -19)	4.85	448
R. PHC	(-33, -40, -12)	4.69	324
R. subiculum	(-17, -13, -22)	4.07	48
L. perirhinal cortex	(31, -13, -28)	4.15	37
R. PHC	(-32, -27, -27)	3.86	25
R. perirhinal cortex	(-35, -12, -30)	3.29	18

Table presents positions and magnitudes of local *t*-statistic maxima, as well as the extent of the activation. Inference is drawn based on an uncorrected threshold *α < 0.01*, two-tailed. The cluster labeled right subiculum also extends into DG/CA3 and partially into CA1.

## Discussion

The present study demonstrated that the PHC and PrC as well as hippocampal subregions subiculum, CA1, and CA3/DG exhibited activation during the DMS sample period that persisted into the delay period in the absence of continued stimulus input. This pattern of observed activation during a WM task was modulated by later subjective memory strength. These results suggest this activity may be reflective of ongoing mechanisms that support encoding of long-term information. In addition, our results suggest ongoing encoding-related activity may slowly decay across the delay period. We discuss theoretical implications of our data in the following sections.

### A Decay Model of Ongoing Encoding

The *decay* model of ongoing encoding expresses the hypothesis activity may be driven initially by the sample period that then persists into the delay waning over the duration of the delay period. This slow decay of persistent activity could reflect the attrition of neuronal firing or attenuation of firing rates. Electrophysiological data has shown that neuronal persistent spiking duration is heterogeneous, ranging from a few seconds (Fraser and MacVicar, 1996), to 10s of seconds (Knauer et al., 2013), to up to several minutes (Yoshida et al., 2008). In addition, slice recording studies have shown some cells exhibit self-terminating persistent firing (Jochems and Yoshida, 2013). While it is impossible to detect single neuron spiking activity with fMRI due to the nature of the BOLD signal, our work suggests that signal may decay over the period of the delay. From this we conclude strong evidence of activity persisting into the delay period in the absence of sensory input. We note this finding is consistent with those of Ranganath et al. (2005), who suggested early (but not late) delay period activity predicts subsequent memory. Originally, we set out to test the hypothesis that the magnitude of sample stimulus driven activity would persist throughout the delay period at a stable level without decay. We also tested the alternate hypothesis that increases in signal could reflect only sample period activity. We examined the fit of these models and found that, although they may each capture different aspects of the data, they are both incompatible with the observed pattern of activity. We therefore revised our initial hypotheses to incorporate a third intermediate model. Together, our simulations show that our ongoing signal is not simply driven by transient activity that ceases when stimulus input disappears, nor is the persistent activation pattern maintained at the same level throughout the sample and delay periods. We suggest while the MTL memory system can maintain stimuli for encoding, this sustained activity pattern may not be solely reflective of a WM maintenance process.

### Medial Temporal Lobe Contributions to Working and Long-Term Memory

Recently, the classical theory of a functional and anatomical dissociation between WM processes (supported by the neocortex) and LTM processes (supported by the MTL; e.g., Shallice and Warrington, 1970), has come into question (see Ranganath and Blumenfeld, 2005; Hasselmo and Stern, 2006 for reviews). Accumulated evidence from human patient studies and animal models supports a critical role for the MTL memory system in normal performance on WM tasks with large sets of novel stimuli. In particular, data suggests amnesic patients with organic hippocampal lesions and those with more extensive damage to parahippocampal regions are impaired relative to controls on delayed match and non-match to sample tasks with novel stimuli even over delay periods as short as a few seconds (Aggleton et al., 1992; Buffalo et al., 1998; Nichols et al., 2006; Race et al., 2013). Targeted lesion studies in monkeys have shown severe impairments on these tasks using novel object stimuli when ablations are restricted to various components of the MTL, especially the PrC (Gaffan and Murray, 1992; Meunier et al., 1993; Zola-Morgan et al., 1993; Eacott et al., 1994). Similarly, in rats, Farovik et al. (2010) showed that lesions of the hippocampal CA1 and CA3 regions induce severe impairments on a non-spatial test of short-term memory for sequences of odors. Studies of trace eyeblink conditioning in mice (Gruart et al., 2014) and rabbits (Carretero-Guillén et al., 2013) showed the importance of temporal hippocampal circuit activation in support of associative learning. This body of literature, together with our findings and complementary human neuroimaging studies that show MTL recruitment during WM tasks in healthy subjects (Ranganath and D’Esposito, 2001; Stern et al., 2001; Schon et al., 2004; Preston et al., 2010; Newmark et al., 2013), implicates the MTL in maintaining neural representations of novel stimuli in WM.

Supported by studies of the electrophysiological properties of neurons in medial temporal cortex, these data have led to the theory that common physiological mechanisms could underlie WM maintenance and long-term episodic encoding (see Ongoing Encoding is Consistent with Persistent Spiking Mechanisms in MTL). Here, we tested the hypothesis that novel stimuli could be continuously encoded into long-term memory over the entire duration they are maintained in WM. This idea of “ongoing encoding” has led us to model sample and delay periods together as one continuous process. Our results support the idea that MTL regions are important during WM for novel stimuli (Ranganath and D’Esposito, 2001; Stern et al., 2001; Schon et al., 2004, 2013; Hasselmo and Stern, 2006). Moreover, we extend previous work by providing evidence that this sustained activity decays on average over a period of several seconds. In addition, the magnitude of the activation pattern linearly predicted how strongly stimuli were encoded. The linear modulation we observed in regard to subsequent memory further suggests that this continued activity reflects ongoing encoding of stimuli.

### Ongoing Encoding is Consistent with Persistent Spiking Mechanisms in MTL

Previous neuroimaging studies, including our own, have interpreted WM delay period activity in the MTL as a possible continuation of encoding (Schon et al., 2004, 2005; Ranganath et al., 2005; Nichols et al., 2006; Olsen et al., 2009), but these studies have modeled the sample and delay separately, reflecting stimulus encoding and short-term maintenance, whereas here we model the sample and delay together to examine the concept of ongoing encoding. The current study also extends these previous whole brain fMRI studies by examining hippocampal subregions using high resolution fMRI methods. Inspiration for our modeling of the fMRI data came from computational frameworks of persistent spiking mechanisms that underpin episodic encoding of novel information (see Hasselmo and Stern, 2006 for a review). Neuronal spiking activity persisting longer than the presence of sensory input may act as a way to hold relevant information, which may, in turn, support successful encoding (Hasselmo et al., 2002; Koene et al., 2003). Initial evidence of persistent spiking activity in the MTL of rodents was found via slice recording data in the EC (Klink and Alonso, 1997; Egorov et al., 2002; Tahvildari et al., 2007; Yoshida et al., 2008). Persistent spiking has since been observed *in vivo* during a delay period of delayed (non-) matching tasks in awake behaving rodents (Young et al., 1997; Deadwyler and Hampson, 2004) and primates (Suzuki et al., 1997). Recent studies have demonstrated subsets of neurons in the PrC (Young et al., 1997; Navaroli et al., 2012) and hippocampal subfields CA1 (Sheffield et al., 2011; Knauer et al., 2013) and CA3 (Jochems and Yoshida, 2013) exhibit persistent spiking, suggesting these regions may also support ongoing encoding via persistent spiking mechanisms. Compatible with this idea, we observed a persistent BOLD signal in PrC, and in the subiculum that extended into CA1 and CA3/DG, however, this signal was most substantial in PHC (**Table 1**; **Figure 4**). Our results suggest the PHC exhibits patterns of persistent activity similar to our other ROIs. As such, we suggest future research should examine electrophysiological properties of neurons in the postrhinal cortex (homolog to primate PHC) to probe for persistent spiking mechanisms.

The slice recording studies we cite above arrive at the conclusion that, in the presence of muscarinic or metabotropic glutamatergic modulation, intrinsic cellular mechanisms involving calcium activated cation currents are sufficient to produce persistent spiking (Shalinsky et al., 2002; Yoshida et al., 2008). While persistent neuronal activity has long been hypothesized to be a cellular mechanism for WM maintenance (Goldman-Rakic, 1995; Wang et al., 2013), other mechanisms could support persistent cellular activation (see Durstewitz et al., 2000 for a review). For example, recurrent network excitation may be able to maintain persistent activity in a neural network (Amit et al., 1994; Amit and Brunel, 1997). In addition, transient cell assemblies may be formed by local circuit mechanisms involving gamma frequency oscillations between pyramidal cells and inhibitory interneurons (Tort et al., 2007). These circuit dynamics could be observed in the MR signal as the BOLD response is correlated with local field potentials in the gamma band (Logothetis et al., 2001). For the present study, we interpret our data within computational frameworks that suggest intrinsic cellular mechanisms, such as persistent spiking, may be necessary to support WM for novel stimuli (Durstewitz et al., 2000; Hasselmo and Stern, 2006).

Ongoing activation in bilateral PHC and PrC was also modulated by subjective memory strength. This is consistent with neuroimaging studies that show involvement of the PHC and PrC during encoding of novel stimuli as well as studies that show sustained activity over short and longer delays (Schon et al., 2004, 2005; Gonsalves et al., 2005; Ranganath et al., 2005; Montaldi et al., 2006; Nichols et al., 2006; Axmacher et al., 2007; Lehky and Tanaka, 2007; Kirwan et al., 2008; Shrager et al., 2008; Olsen et al., 2009; Preston et al., 2010; Song et al., 2011). In monkeys, lesions of the PrC and PHC severely impair performance on a delayed non-match to sample task (Suzuki and Zola-Morgan, 1993), and an fMRI study implicated the PrC and the hippocampus in successful encoding measured by SMTs (Miyamoto et al., 2014). Our PHC coordinates are consistent with prior studies that localize the parahippocampal place area (Epstein et al., 1999). This region has been implicated in processing of scene stimuli and contextual associations more generally (Aminoff et al., 2007; Epstein et al., 2007; Awipi and Davachi, 2008; Bar et al., 2008; Preston et al., 2010; Turk-Browne et al., 2012). Since we used complex outdoor scenes as stimuli, this congruity is not surprising. Our results extend the involvement of the PHC from contextual processing to ongoing encoding.

Our results also demonstrate that persistent activity in the right hippocampal subfields CA1, CA3/DG, and the subiculum was related to memory strength. Previous high-resolution neuroimaging studies have examined functional dissociations between hippocampal subfields, including studies examining subregional involvement in pattern separation and pattern completion (see Yassa and Stark, 2011 for a review). Recent work from our lab has shown that subregions CA1 and subiculum are important for disambiguation of overlapping input in a WM task (Newmark et al., 2013). Although our DMS task was not designed to examine pattern separation/disambiguation, encoding of our content-similar stimuli may have required participants to disambiguate the similarities across scenes. As such, our findings are consistent with our earlier results (Newmark et al., 2013) that suggested increased CA3/DG recruitment during the sample phase, and increased CA1 and subiculum recruitment during a delay period of a WM task.

## Conclusion

The current study provides evidence that increased recruitment of the PHC, PrC, and hippocampal subfields CA1, CA3/DG, and subiculum are associated with ongoing encoding and subsequent memory strength. Based on the fit of various encoding models we conclude that activity related to sample stimuli persists following stimulus cessation, but that this activity may, on average, gradually taper off throughout the delay. In addition, the magnitude of the activation pattern linearly predicted how strongly stimuli were encoded. Our results support theories formalized by computational models that common processes within the MTL could drive both long-term encoding and WM maintenance.

## Conflict of Interest Statement

The authors declare that the research was conducted in the absence of any commercial or financial relationships that could be construed as a potential conflict of interest.

## Footnotes

1 http://cibsr.stanford.edu/tools/human-brain-project/artrepair-software.html

2 http://www.itksnap.org/

3 http://picsl.upenn.edu/software/ants/
